# Efforts in favor of health

**Published:** 2014

Undoubtedly, the initiative of the demarches which are responsible, visible and benefic for “Health-Nutrition-Wellbeing” belong to Prof. Florian Popa, MD, Vice-president of the Health Commission of the Senate of Romania and the President of the National Council of Attesting the Titles, Diplomas and University Certificates in Medicine, at that time, Rector of “Carol Davila” University of Medicine and Pharmacy in Bucharest, in the first mandate.

**Fig. 1:**
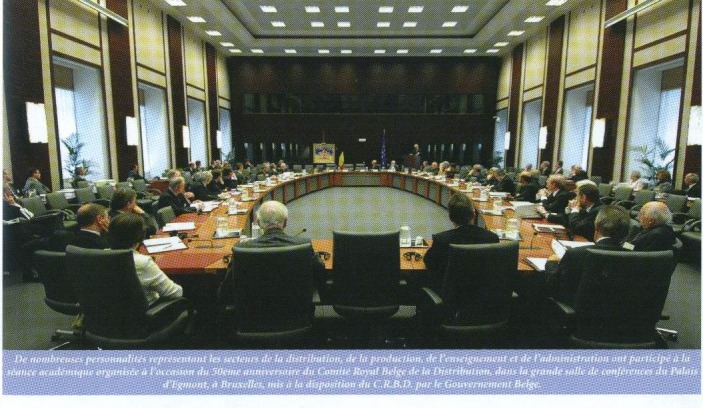
“50-eme Anniversaire du Comite Royale Belge de la Distribution”

However, it all started from the meeting on November 23, 2004 on which occasion the **“50-eme Anniversaire du Comite Royale Belge de la Distribution”** took place.

The meeting took place at the Royal Palace in Brussels and had as guests many political and European academic personalities. The discussions had as a main theme health and nutrition and have culminated, nine years later, on the 15th of February 2013, obviously, in the presence of Prof. Florian Popa but also of other important diplomatic, political and academic personalities, with the awarding of the **“Golden Archer” – The Great Prize for Innovation of Belgium**, to a valuable group of “Carol Davila” University of Medicine and Pharmacy in Bucharest and the leader of this group, in Brussels, at Saint Anne Castle, the Belgian Diplomacy Club.

**Fig. 2:**
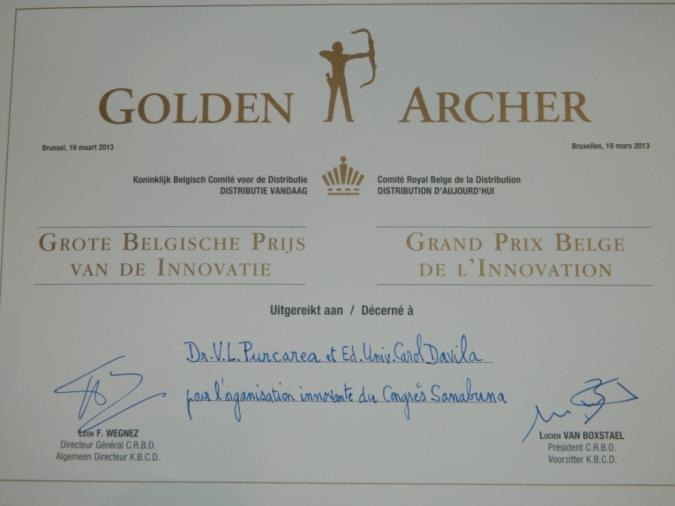
Golden Archer Diploma

**How did we get here?**

What followed was the First National Conference “Health-Nutrition-Wellbeing”, at “Carol Davila” University of Medicine and Pharmacy in Bucharest in 2009, the First International Congress “Health-Nutrition-Wellbeing” in Brasov, in 2011 and the Second International Congress “Health-Nutrition-Wellbeing”, in Falticeni, in 2012.

The First National Congress “Health-Nutrition-Wellbeing” was the expression of responsibly assuming the partnership for “nutrition – health – wellbeing” and the restoration of trust in life and on the market. The prestigious event approached the imperative of reflection and also actions regarding the issue of “health-nutrition-wellbeing”, trying to identify a new necessary direction of attitudes which will allow the foundation of a public-private partnership which will be able to offer adequate solutions of influencing the behavioral change in order to improve the health and the economical-social responsibility.

From the very first beginning of the works of **SANABUNA 2009 Conference**, the following pertinent interventions took place: *Florian Popa* (Member of the Academy of Medical Sciences and Rector of “Carol Davila” University of Medicine and Pharmacy in Bucharest), *Laurentiu Mircea Popescu* (President of the Academy of Medical Sciences and President of the Medical Department of the Romanian Academy), *Theodor Valentin Purcarea* (President of the Romanian Distribution Committee, Member of A.I.D.A. Council in Brussels, Professor in the Romanian-American University), Assoc. Prof. Dr. Eng. *Victor Surdu *(Deputy, Member of the Romanian Parliament), *Constantin Opran* (Rector of “Lucian Blaga” University in Sibiu),, Acad. *Constantin Popa *(Chief of Internal Medicine Department of the Academy of Medical Sciences), *Prof. A.V. Ciurea*, MD (Vice-president of the World Federation of the Societies of Neurosurgery – WFSN, President of Honor of the Romanian Society of Neurosurgery), *Bogdan Dumitrescu* (Sommelier), shareholders of some important private companies.

**Fig. 3:**
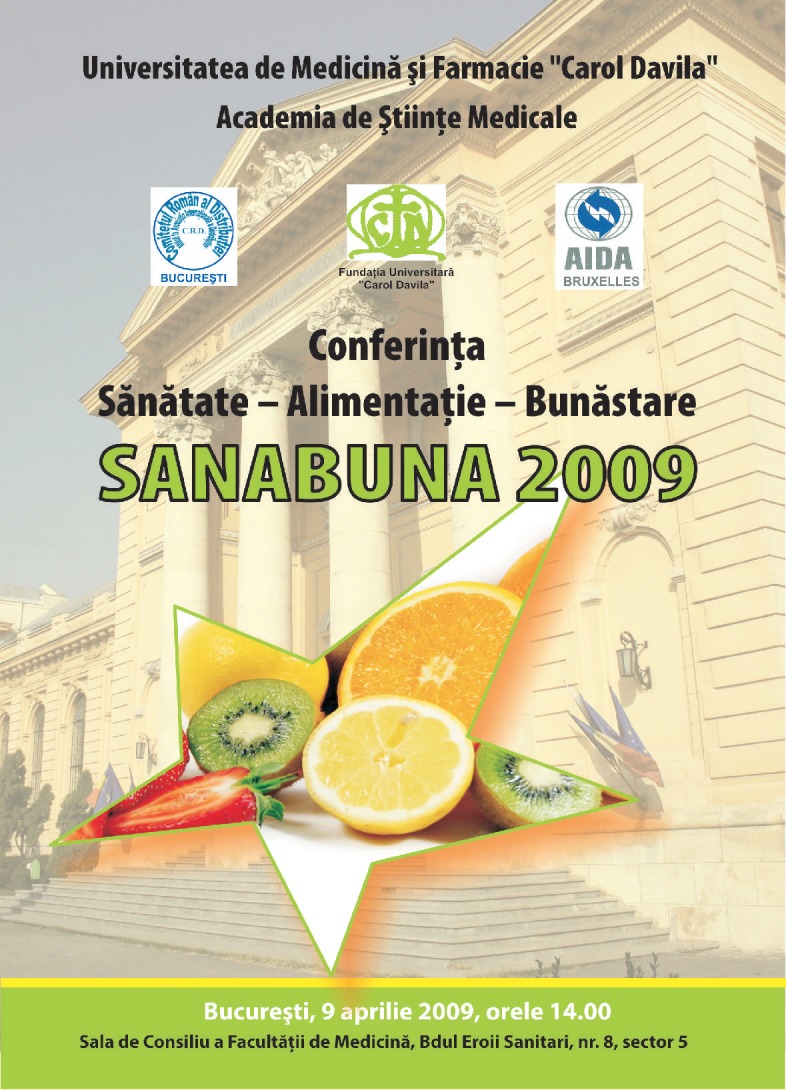
SANABUNA 2009 – event banner

*Mass media has reflected* SANABUNA 2009 Conference *with its well-recognized professionalism.* This way, *“Romanian Wine Art”* magazine, issue 30, April-May 2009 described the event in a few words, in an article named “SANABUNA 2009: at the confluence of the preoccupations for a better life” - the important evidence of this responsible enrolment in the partnership for “nutrition – health – wellbeing”. Moreover, *“Sanatatea TV”* (http://www.sanatateatv.ro/stiri-medicale/prof-dr-florin-popa-schimbarea-alimentatiei-unui-popor-este-mai-grea-decat-o-revolutie/) also underlined the special signification of the issues approached in “SANABUNA 2009” Conference, in the context of the lack of trust crisis, health being the first of all freedoms.

**Fig. 4:**
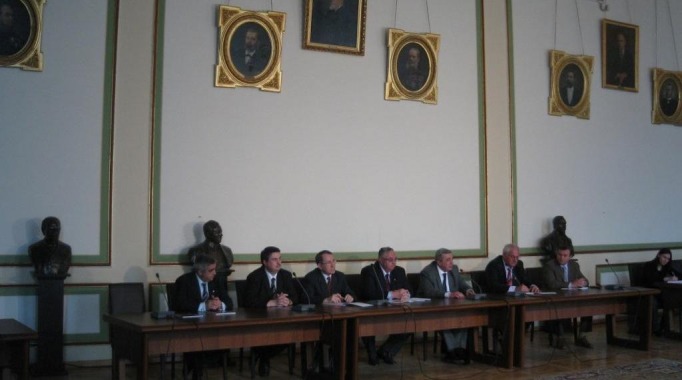
Debates regarding health, nutrition and wellbeing in “Carol Davila” University of Medicine and Pharmacy, Bucharest, Council Hall

The interdisciplinary character of the manifestation allowed the covering of some aspects of real topicality by building and sustaining the trust of the consumer in the nutrition chain and by the manner of directing the best minds for a sustainable “Health-Nutrition-Wellbeing” relationship, making a current cultural preoccupation out of this.

The pertinent interventions during the debates (from which we evidence the one belonging to Florian Popa, Rector of “Carol Davila” University of Medicine and Pharmacy, Bucharest; Laurentiu Mircea Popescu, President of the Academy of Medical Sciences; Theodor Valentin Purcarea, President of the Romanian Distribution Committee, President of A.I.D.A. Romania, Professor in the Romanian-Americal University; Constantin Oprean, Rector of “Lucian Blaga” University in Sibiu; Acad. Constantin Popa, and Victor Surdu, member of the Romanian Parliament) evidenced the importance of the partnership in the interaction between the nutritional lifestyle and the other lifestyle factors.

The health of the people is the center of sustainable development and of adapting the business itself.

A wine tasting action also took place at the end of the manifestation. The wines which were tasted came from famous vineyards: Dealu Mare- IDLE ROCK- and Ceptura- ROTEMBERG. They were presented by a sommelier and were very well welcomed by the participants.

SANABUNA 2009 message was clear: to recognize the moment of truth and to invite the architects of conversation which generates responsible actions to team working, while taking into account the fact that the “therapist” exists in each person who responsibly engages in the “nutrition-health-wellbeing” partnership, which can restore trust in life and in the market.

The First International Congress **„Health-Nutrition-Wellbeing”**, just like we presented in detail in the pages of our journal, took place in Aro Palace Hotel, Brasov, on 15-17 October 2011, under the patronage of the ***Romanian Patriarchy***, the ***Romanian Academy***, the ***Ministry of Public Health***, the ***Ministry of Agriculture and Rural Development*** and the*** Ministry of Education, Research, Innovation and Sports***, in collaboration with the ***International Distribution Association (A.I.D.A. Brussels***) and the ***European Retail Institute EHI (Institute of European Commerce*)** in ***Köln***.

**The organizers** were “Carol Davila” University of Medicine and Pharmacy, Romanian-American University, “Lucian Blaga” University in Sibiu, “Spiru Haret” University, “Transilvania” University in Brasov, Academy of Medical Sciences in Romania, National Agency for Medicines and Medical Devices, Institute of Nutritional Bioresources. 

**Fig. 5:**
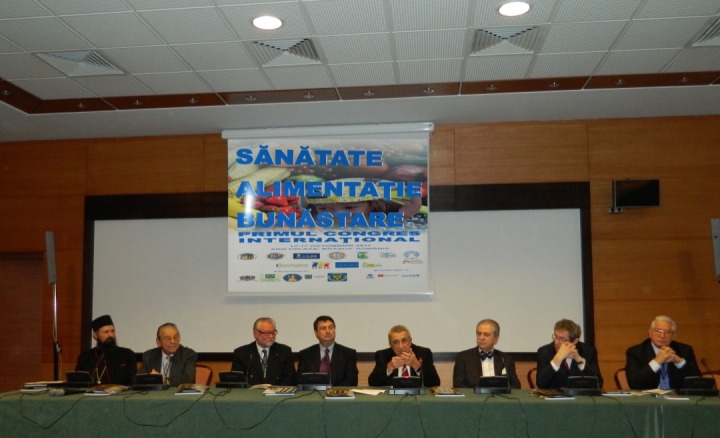
First International Congress “Health-Nutrition-Wellbeing”, Aro Palace Hotel, Brasov, Romania

The following personalities were also part of the presidium: Professor **Florian Popa**, President of the Organizing Committee of the Congress, Rector of “Carol Davila” University of Medicine and Pharmacy in Bucharest, member of the Health Commission of the Deputies Chamber in the Romanian Parliament; the honor presidents of the congress: Professor **Eliot Sorel** from George Washington University, Professor **Bernd Hallier**, Managing Director of the European Commerce Institute in Koln, President of the European Center of Competence for Vocational Formation in Retail, President of the European Academy of Retail and Professor **John L. Stanton** from “St. Joseph” University in Philadelphia, **Professor Adrian Streinu Cercel**, Secretary of State in the Ministry of Public Health and Chancellor of “Carol Davila” University of Medicine and Pharmacy, Professor **Theodor Valentin Purcarea**, from the Romanian-American University, President of the Romanian Distribution Committee, Member in the Council of the International Association of Distribution - A.I.D.A. Brussels, Professor **Ion Petrescu**, Rector of “Spiru Haret” University, in Brasov and Professor **Alexandru Vlad Ciurea**, Secretary General of the Congress.

**Fig. 6:**
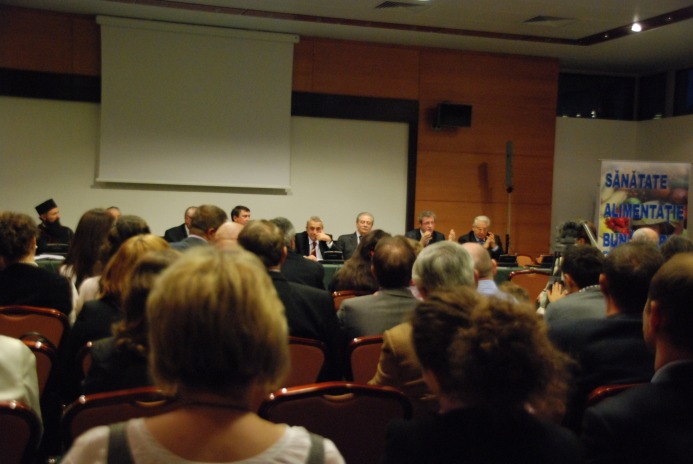
Debates - First International Congress “Health-Nutrition-Wellbeing”

The way the President of the Congress, Prof. Florian Popa, MD, briefly declared in the prestigious **„Journal of Medicine and Life”**(Volume 4, Issue 4, October-December 2011) and also detailed in the **Special Issue** (Vol. 4, Second Edition), on that occasion, a letter of intent (www.european-retail-academy.org/index.php, 17.10.2011: New Global Initiative) was signed, with the purpose of a future cooperation in one of the following fields: a Romanian Center of Competence for Good global agricultural practices, “intelligent agricultural aliments” and the enrichment of the Youth Eurasian Forum with “The initiative of the Black Sea/ Caspic Sea” to promote academic education, innovation and the Bologna system. 

The Second International Congress “Health-Nutrition-Wellbeing”, “SANABUNA International”, which took place in October 2012, in Falticeni, Romania, developed in extremely special conditions. 

The elements which have contributed to the creation of these special conditions were first of all the dream lands of this blessed place, the inexhaustible organizational efforts of some hosts who were very capable and friendly, the overwhelming personality of the honor guests from Romania and from abroad, the distinction and the warmth of some important academic personalities, the opening and the erudition of some political and administrative personalities and, not least, the enthusiasm, the energy and the interest of the participants, among whom, there was a significant amount of students in medicine from seven countries. 

**Fig. 7:**
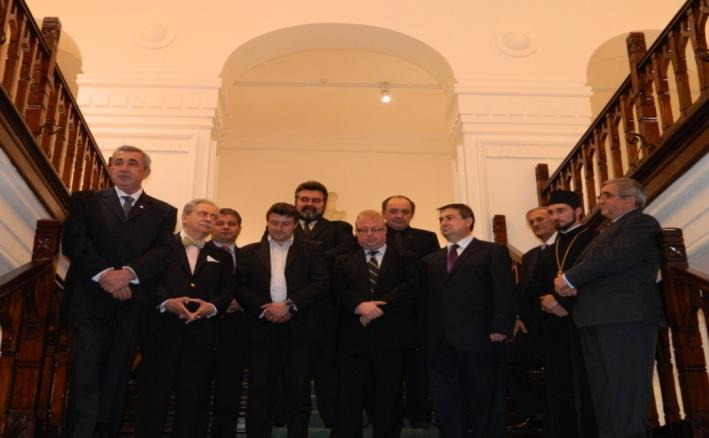
International Congress “Health-Nutrition-Wellbeing” (SANBUNA International), Fălticeni, Suceava

Taking into account the real interest proved in Fălticeni and also in Suceava in promoting the Public-Civic-Private Partnership, while taking into account the whole series of potential benefits: health, social and direct and indirect economical ones, regarding the medium concerning “The Pilot Station” in Fălticeni and also an assumed challenge, “Building an Eastern European Regional Model of Thought and Action “Fălticeni Challenge” and the well-known “Rădășeni Apples” which have enchanted the participants, the message and the action of the International Congress “Health-Nutrition-Wellbeing” (SANBUNA International) has been a clear and motivated urge to open our minds and go beyond the fragments of understanding, by interacting, getting involved, communicating and building a Public-Civil-Private Partnership which can restore the trust in life and in the market, so that the health of people and of the economy remains the center of sustainable development and of adapting the business as a consequence.

To quote the President of the Congress, it represented “a starting point for the reform of wellbeing, because the discourse and the action regarding the problem: “nutrition – health – wellbeing” must and can be modified so as to preserve life by opening our minds, ascending our spirits, overcoming the understanding fragmentations, interacting, getting involved, communicating and learning how to make the adequate change in our behavior of individual and organizational consumers of aliments”. 

**Fig. 8:**
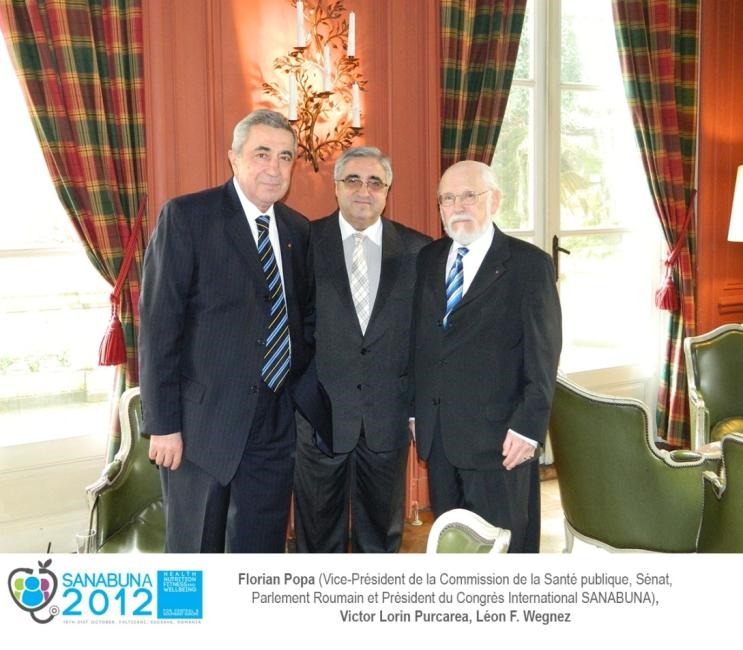
Group picture – SANABUNA 2012

The challenge in confronting the reform of wellbeing, affirmed the President of the Congress, does not lie in defending or opposing the levels of social expense or in the obfuscation of the people as far as the benefits or the specific rules are concerned but by reconfiguring, through consistent ways, with values such as the ones supported by the social European model. 

The necessity of adapting the contemporary economical environment to the changing economical currents and to a modern thinking regarding the role of the social politics in meeting new sources of concurrence due to the technological change or to the different manifestations of globalization, but we should not omit the fact that education is the core of adaptation and solidarity cannot be neglected. The scientific strategies of improving the innovation processes and of stimulating the equality in the food chains are needed; only this way, health, happiness and longevity will be improved and the trust of the consumer in the food chain will be built and sustained.

This special issue has proposed to remind the participants these successful events and useful scientific manifestations, as well as some of the appreciated papers, which, why not, represent “a challenge” for the future ones.

**Executive Editor** **Assoc. Prof. Dr. Eng. Victor Lorin Purcarea**

